# TrkB-Mediated Neuroprotective and Antihypoxic Properties of Brain-Derived Neurotrophic Factor

**DOI:** 10.1155/2015/453901

**Published:** 2015-05-13

**Authors:** Maria V. Vedunova, Tatiana A. Mishchenko, Elena V. Mitroshina, Irina V. Mukhina

**Affiliations:** ^1^Laboratory for Neuroprotection Methods Development, Nizhny Novgorod Neuroscience Centre, Lobachevsky State University of Nizhny Novgorod, Nizhny Novgorod, Russia; ^2^Molecular and Cell Technologies Group, Nizhny Novgorod State Medical Academy, Nizhny Novgorod, Russia

## Abstract

The neuroprotective and antihypoxic effects of brain-derived neurotrophic factor (BDNF) on dissociated hippocampal cultures in a hypoxia model were investigated. These experiments demonstrate that 10 minutes of normobaric hypoxia increased the number of dead cells in primary culture, whereas a preventive application of BDNF increased the number of viable cells. Spontaneous bioelectrical and calcium activity in neural networks was analyzed using multielectrode arrays and functional intravital calcium imaging. The results indicate that BDNF affects the functional parameters of neuronal networks in dissociated hippocampal cultures over the 7-day posthypoxic period. In addition, the effects of k252a, an antagonist of tropomyosin-related kinase B (TrkB), on functional bioelectrical activity during and after acute hypoxia were investigated. It was shown that the protective effects of BDNF are associated with binding to the TrkB receptor. Finally, intravital fluorescent mRNA probes were used to study the role of NF-*κ*B1 in the protective effects of BDNF. Our experiments revealed that BDNF application stimulates NF-*κ*B1 mRNA synthesis in primary dissociated hippocampal cells under normal conditions but not in hypoxic state.

## 1. Introduction

Hypoxia is thought to be one of the main contributors to ischemic tissue damage. Changes in neuronal oxygen metabolism can alter many processes, including synaptic transmission and cell death, leading to the destruction of neural networks in the brain [1, 2]. Brain-derived neurotrophic factor (BDNF) is one factor that can control cellular metabolic rates under low oxygen conditions and promotes neuronal survival [3, 4]. This protein plays an important role in neuronal differentiation and the development of synaptic contacts during neurogenesis, but it can also act as a modulator of mature neuronal metabolism [5–10].

Potential protective mechanisms of mature BDNF relate with the ability of this protein to bind with TrkB-FL receptor, leading to the activation of intracellular signaling cascades [11–13].

One of the signaling pathways associated with cell survival during hypoxia is activation of the nuclear factor kappa-light-chain-enhancer of activated B cells (NF-*κ*B) protein complex. NF-*κ*B type 1 can exist as a homo- or heterodimeric complex that is normally inactive and localized to the cytoplasm via the specific inhibitor I*κ*B (inhibitor of *κ*B) [14]. NF-*κ*B1 responds to the expression of proteins from the Bcl2 (B-cell lymphoma 2) and IAP (inhibitors of apoptosis) families [15]. According to the previous data BDNF can activate NF-*κ*B synthesis [16]. Therefore, increased NF-*κ*B1 mRNA synthesis induced by BDNF may be considered one of the possible mechanisms through cell metabolism alteration as a response to low oxygen conditions.

In this study, we investigate the antihypoxic and neuroprotective properties of BDNF. We examined changes in the spontaneous bioelectrical and calcium activities of neural networks in dissociated hippocampal cultures during acute normobaric hypoxia and the following 7 days.

## 2. Materials and Methods

### 2.1. Ethics Statement

All experimental protocols in this research were reviewed and approved by the Bioethics Committee of Nizhny Novgorod State Medical Academy; experiments were conducted in strict accordance with Act 708n (23.08.2010) the National Ministry of Public Health of Russian Federation approving the rules of laboratory practice for the care and use of laboratory animals and Council Directive 2010/63EU of the European Parliament (September, 22, 2010) on the protection of animals used for scientific purposes. C57BL6J mice were sacrificed by cervical vertebra dislocation. Embryos were then surgically removed and sacrificed by decapitation.

### 2.2. Cell Culture

Hippocampal cells were dissociated from embryonic mice (on embryonic day 18) and plated at a high initial density of approximately 9000 cells/mm^2^ on MEAs (Alpha MED Science, Japan) or coverslips pretreated with the adhesion-promoting molecule polyethyleneimine (Sigma P3143, Germany). Hippocampi were cut in Ca^2+^- and Mg^2+^-free phosphate-buffered saline (PBS-minus), followed by enzymatic digestion for 25 min with 0.25% trypsin (Invitrogen, 25200-056, USA). After carefully suspending the cells, the solution was centrifuged at 1000 ×g for 3 min, and the cell pellet was immediately resuspended in Neurobasal medium (Invitrogen, 21103-049, USA) with 2% B27 (Invitrogen, 17504-044, USA), 0.5 mM L-glutamine (Invitrogen, 25030-024, USA), and 5% fetal calf serum (PanEco K055, Russia). The dissociated cells were seeded onto MEAs or coverslips. After 24 h, the medium was replaced with Neurobasal medium with 2% B27, 1 mM L-glutamine, and 0.4% fetal calf serum. One-half of the medium was changed every 2 days. The cells were cultured under constant conditions of 35.5°C and 5% CO_2_ at saturating humidity in a cell culture incubator [17].

### 2.3. Hypoxia Model

Hypoxia modeling was performed on day 14 of culture development* in vitro* (DIV) by replacing the normoxic cultural medium with a medium containing low oxygen for 10 min. The oxygen was displaced from the medium in sealed chamber in which the air was replaced with an inert gas (argon). The oxygen concentration decreased from 3.26 mL/L (normoxia) to 0.37 mL/L (hypoxia) [18, 19].

All cultures were divided into the following experimental groups: (1) BDNF (1 ng/mL) (Millipore, GF029, USA) (*N* = 9); (2) k252a (150 nM) (Sigma, K2015, Germany), an inhibitor of the Trk receptors (*N* = 9); and (3) BDNF (1 ng/mL) plus k252a (150 nM) (*N* = 9) added to the medium 20 min before hypoxia. In the control group (*N* = 9), hypoxia was induced without additional treatment.

### 2.4. Electrophysiological Methods

Extracellular potentials were recorded simultaneously using 64 planar indium tin-oxide (ITO) platinum black electrodes with the integrated MED64 system (Alpha MED Science, Japan). MEAs were arranged in an 8 × 8 array (64 total) with a 50 × 50 *μ*m electrode size, 150 *μ*m spacing and a sampling rate of 20 kHz/channel. All of the signal analyses and statistics were performed using custom-made software (Matlab).

We recorded spontaneous bursting activity for 10 min. To detect small bursts, we calculated the total spiking rate (TSR) accounting for the total number of spikes from all electrodes within 50 ms time bins. The rapid appearance of a large number of spikes over multiple electrodes within a small (50 ms) time bin was used as the criterion for small bursts [17, 20].

#### 2.4.1. Spike Detection

The detection of recorded extracellular spikes was based on threshold calculations using the signal median:

(1) $$\begin{array}{c} T=N_{S}\sigma ,\\ \sigma =\text{median}\left(\frac{\left|x\right|}{0.6745}\right), \end{array}$$

where *x* is the band-pass-filtered (0.3–8 kHz) data signal, *σ* is an estimate of the standard deviation of the signal without spikes [21], and *N*_*S*_ is a spike detection coefficient determining the detection threshold [20]. During signal processing, threshold estimations based on the median of the signal in the form of (1) are less dependent on the frequency of the spikes than are estimations based on standard deviation. Coefficient 0.6745 in (1) is used to normalize the median of the absolute signal to the standard deviation. *N*_*S*_ = 4 was used for all data, allowing the reliable detection of spikes with amplitudes greater than 20 *μ*V. The minimal interspike interval was set to 1 ms. Detected spikes were plotted using raster diagrams.

#### 2.4.2. Small Burst Detection

To analyze the effect of BDNF on neural network activity, we recorded spontaneous bursting activity for 10 min. To detect small bursts, we calculated the TSR by accounting for the total number of spikes from all electrodes within 50 ms time bins. The fast appearance of a large number of spikes over multiple electrodes within a small (50 ms) time bin was used as the criterion for small bursts (for more details, see Pimashkin et al., 2011). Spontaneous activity in the cultures consisted of basal stochastic activity observed in fractions of cells together with short bursting episodes. Basal activity consisted of a spike trains (approximately 1 spike per 10–100 ms). To detect bursts, we used threshold detection based on the statistical characteristics of the spontaneous activity TSR(t). Burst threshold was set to TBurst = 0.1 × *σ*TSR, where *σ*TSR is the standard deviation of TSR(t). To exclude basal activity, the burst detection threshold coefficient was empirically set to 0.1, giving the best estimate for the burst initiation and end points according to the raster diagram. Simulations of bursts with frequencies up to 5 Hz revealed that the estimated burst durations were within 10% of the real values. Statistical analysis of the bursting activity characteristics was performed using analysis of variance (ANOVA) (*p* < 0.05) [17].

### 2.5. Ca^2+^ Imaging

To conduct functional calcium imaging, Oregon Green 488 BAPTA-1 AM (OGB-1) (0.4 *μ*M, Invitrogen, O-6807, USA) was dissolved in dimethylsulfoxide (DMSO) (Sigma, D8418, Germany) with 4% Pluronic F-127 (Invitrogen, P-3000MP, USA) and then added to the culture medium for 40 min. After incubation to allow the OGB-1 molecules to be fully absorbed by the cells, the cells were washed for 15 min with dye-free medium. A confocal laser-scanning microscope (Zeiss LSM 510, Germany) with a W Plan-Apochromat 20×/1.0 objective was used to visualize spontaneous calcium activity in the dissociated cultures.

Cytosolic Ca^2+^ was visualized via OGB-1 excitation with 488 nm Argon laser excitation and emission detection with a 500–530 nm filter. Time series of 256 × 256 pixel images of 420 *μ*m × 420 *μ*m fields of view were recorded at 4 Hz. A confocal pinhole of 1 airy unit was used to obtain an axial optical slice resolution of 1.6 *μ*m.

Quantitative evaluation of Ca^2+^ transients was performed off-line using custom-made software in C++ Builder. Cell regions from fluorescent images were manually selected. The Ca^2+^ fluorescence of each cell in each frame was calculated as the average fluorescence intensity (*F*, relative units from 0 to 255) of the pixels within the defined cell region. Single Ca^2+^ signals were identified using the following algorithm. First, each trace from all of the cells was filtered by averaging two neighboring points in the sample set. Next, we calculated a simple derivative of the signal by determining the difference between each pair of consequent points. The pulses were identified from the derivative of the trace using a threshold detection algorithm. The threshold was estimated as the detection accuracy coefficient multiplied by the standard deviation of the derivative of the trace. Suprathreshold points on the derivative of the trace were taken as the beginnings and ends of the pulses [17, 22].

### 2.6. mRNA Detection

We studied intravital RNA expression in cells using SmartFlare RNA Detection Probes (Merck Millipore, France) and fluorescent microscopy. The RNA Detection Probes are absorbed by living cells via endocytosis. These probes circulate within the cell and bind to complementary target RNA sequences. This binding event releases a fluorophore that can be detected by fluorescence microscopy. SmartFlare probes for the NF-*κ*B1 mRNA with the Cy5 fluorophore (Merck Millipore SF-745, France) were diluted in 50 *μ*L sterile apirogen water to create a working solution. Then, 0.5 *μ*L diluted SmartFlare reagent was added to each cell culture dish, followed by incubation in a culture incubator for 16 hours. The following controls were used: (1) uptake control (positive control) (Merck Millipore SF-137, France), which is always fluorescent inside cells; (2) scramble control (negative control) (Merck Millipore SF-102, France), which does not recognize any mRNA sequences within cells and was used to assess cell autofluorescence; and (3) housekeeping probe (Merck Millipore SF-142, France) is a positive control for mRNA detection and is always active in cells (detects mRNA 18S). A confocal laser-scanning microscope (Zeiss LSM 510, Germany) was used to visualize mRNAs via Cy5 excitation with the 633 nm line of a He-Ne laser and emission detection with a 650–710-nm filter.

### 2.7. Cell Viability Detection

The viability of dissociated hippocampal cells was evaluated according to the percentage ratio between the number of dead cells stained by propidium iodide (Sigma, P4170, Germany) and the total number of cells stained by bisBenzimide (Invitrogen, H3570, USA) for 7 days after hypoxia [18, 19].

### 2.8. Statistical Analysis

All quantified data are presented as the mean ± standard error of the mean (SEM) values. Statistical analyses were performed using two-way ANOVA implemented in the Sigma Plot 11.0 software program (Systat Software, Inc.). Student-Newman-Keuls (SNK) was used as a post hoc ANOVA test. Differences between groups were considered significant if the corresponding *p* value was less than 0.05.

## 3. Results

First, we investigated the main parameters of spontaneous bioelectrical activity in dissociated hippocampal cultures. Previous studies demonstrated the appearance of spontaneous bioelectrical activity in dissociated hippocampal cultures during days 8–10 of development using multielectrode arrays [17, 18]. The activity of primary cultures had stabilized by DIV 14.

**Figure 1:**
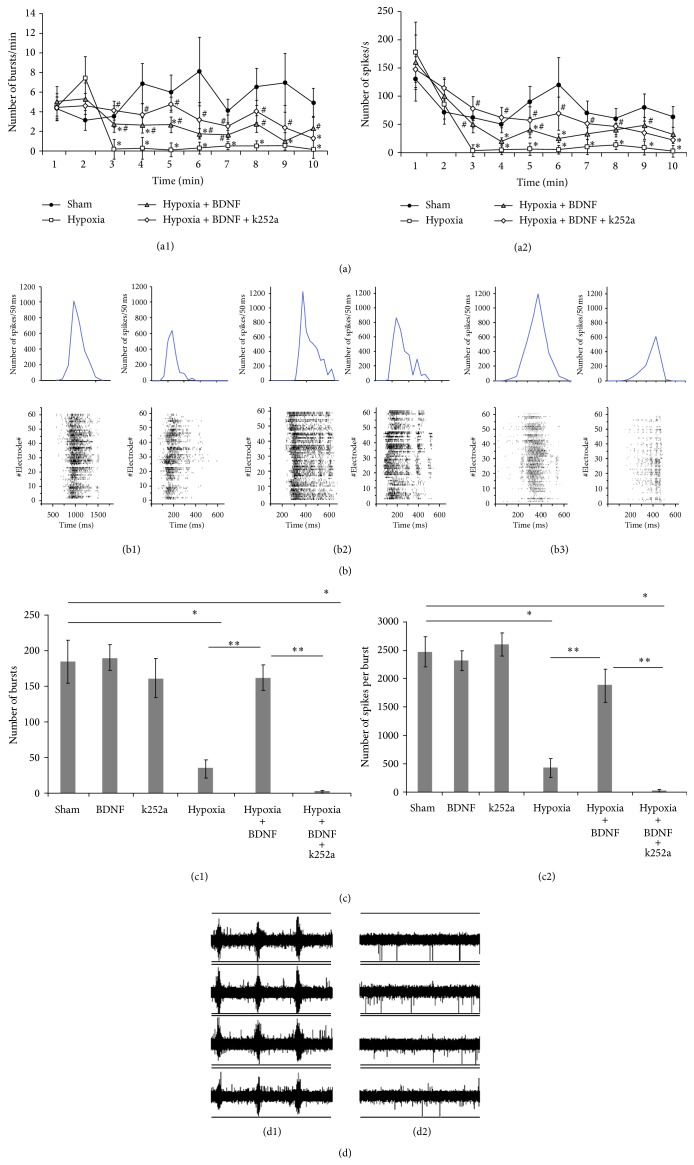
Spontaneous bioelectrical activity in dissociated hippocampal cultures. ((a1)-(a2)) Neural network activity during the first 10 min of hypoxia. (a1) number of network bursts per min; (a2) number of spikes per sec; ((b1)–(b3)) Raster plots of electrical spiking activity from 64 electrodes (lower rows) and a diagram of the total spike rate (upper row) in primary hippocampal cultures. (b1) hypoxia; (b2) hypoxia + BDNF; (b3) hypoxia + BDNF + k252a. ((c1)-(c2)) Neural network activity on day 7 after hypoxia; (c1) number of network bursts per 10 min; (c2) number of spikes per burst; ((d1)-(d2)) spike recording from 4 electrodes of the MED64 probe. (d1) before hypoxia; (d2) on 7 day after hypoxia. (∗: versus “Sham”; ∗∗: versus “hypoxia + BDNF”; #: versus “hypoxia”, ANOVA *p* < 0.05, *N* = 9).

Hypoxia modeling was performed on the 14th day of culture development. We observed an inhibition of spontaneous bioelectrical activity starting at the third minute of hypoxia (Figures 1(a1)-(a2)) (Sham: 3.54 ± 1.13 bursts/min, Hypoxia: 0.21 ± 1.09 bursts/min; Sham: 62.21 ± 18.64 spikes/s, Hypoxia: 3.04 ± 10.53 spikes/s; ANOVA *p* < 0.05; *N* = 9). Reoxygenation caused an increase the neuronal network activity compared with baseline measurements. The pattern of spontaneous bioelectrical activity changed with respect to the number of small bursts (before hypoxia 38.20 ± 7.83 bursts/min, after hypoxia: 101.70 ± 23.67 bursts/min; ANOVA *p* < 0.05; *N* = 9), and we observed an insignificant increase in the average number of spikes per burst (before hypoxia: 251.82 ± 102.14 spikes/burst, after hypoxia: 432.71 ± 141.36 spikes/burst, *N* = 9). However, there was an irreversible decrease in the spontaneous bioelectrical activity 2 hours after hypoxia (3.00 ± 5.67 bursts; average 508.00 ± 232.98 spikes/burst, *N* = 9).

It was shown that the preventive application of BDNF (1 ng/mL) in culture medium can partially preserve neuronal activity during hypoxia (Figure 1(a1)-(a2)) (hypoxia: 0.21 ± 1.02 bursts/min, hypoxia + BDNF: 2.78 ± 0.83 bursts/min; hypoxia: 3.02 ± 10.54 spikes/s, hypoxia + BDNF: 50.05 ± 15.12 spikes/s; ANOVA *p* < 0.05; *N* = 9). A combination of the TrkB receptor inhibitor k252a (150 nM) and BDNF (1 ng/mL) could also partially preserve spontaneous bioelectrical activity during hypoxia. However, network activity in this group was irreversibly disrupted 2 hours after reoxygenation (2.31 ± 4.96 bursts in 10 min; average 214.32 ± 112.41 spikes/burst, *N* = 9). The obtained data are confirmed by the previous experimental studies [18, 19].

An analysis of spontaneous bioelectrical activity performed the day after hypoxia revealed significant changes in the pattern of activity and a partial disruption of burst activity in the hypoxia group and in the cultures treated with k252a (150 nM) (Figure 1(b)). This destruction of functional network activity was observed for 7 days after the hypoxic period. By the 7th day of the posthypoxic period, there was an almost complete loss of network activity (Figure 1(d1)-(d2)). However, treatment with BDNF (1 ng/mL) contributed to maintenance burst activity during this period (Figure 1(c1)-(c2)) (Sham: 185.03 ± 30.33 bursts, hypoxia + BDNF: 163.34 ± 18.01 bursts; average Sham: 2473.57 ± 267.42 spikes/burst, hypoxia + BDNF: 1876.66 ± 278.73 spikes/burst, *N* = 9).

Blocking TrkB receptor activity completely negated the positive effects of BDNF treatment (Figure 1(c1)-(c2)) (hypoxia + BDNF: 163.24 ± 18.15 bursts, hypoxia + BDNF + k252a: 3.35 ± 1.27 bursts; average hypoxia + BDNF: 1876.11 ± 278.25 spikes/burst, hypoxia + BDNF + k252a: 35.73 ± 11.08 spikes/burst; ANOVA *p* < 0.05; *N* = 9). Application of k252a alone also altered spontaneous bioelectrical activity relative to the hypoxia group (7 days posthypoxia: 11.37 ± 7.46 bursts; average 82.87 ± 76.31 spikes/burst, *N* = 6). The single application of BDNF as well as k252a on the culture medium did not significantly affect the main parameters of spontaneous bioelectrical activity of dissociated hippocampal cultures under normal oxygen conditions (Figures 1((c1)-(c2))).

**Figure 2:**
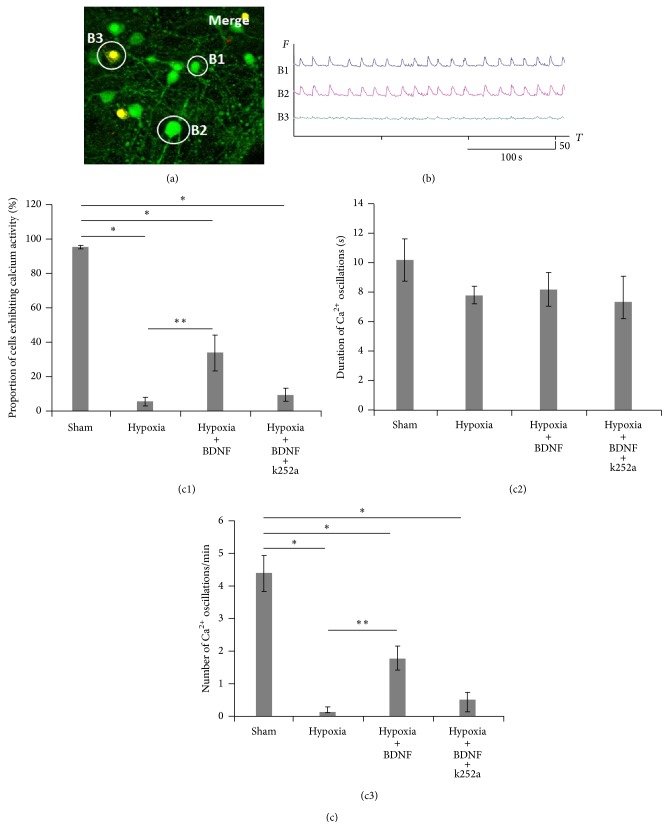
Spontaneous calcium activity in dissociated hippocampal cultures in the posthypoxic period. (a) Example of a frame from the calcium-dependent fluorescent dye image series; green, cells marked with Oregon Green; yellow, propidium iodide-positive cells. (b) Spontaneous Ca^2+^ oscillation recordings in marked neurons. (B1-B2) Ca^2+^ oscillations in cells stained with Oregon Green; (B3) Ca^2+^ oscillations in cells stained with propidium iodide. ((c1)–(c3)) The main parameters of calcium activity in dissociated hippocampal cultures after 7 days of hypoxia. (c1) proportion of cells exhibiting calcium activity; (c2) duration of Ca^2+^ oscillations; (c3) number of Ca^2+^ oscillations per min (ANOVA *p* < 0.05, *N* = 12).

Next, we investigated spontaneous calcium activity in dissociated hippocampal cultures. Previous studies demonstrated the appearance of spontaneous calcium oscillations in dissociated hippocampal cultures from DIV 7. By the 14th day of development, large numbers of neurons showed similar patterns of calcium activity (Figure 2(a)). This phenomenon of calcium activity was fairly stable and did not significantly change up to DIV 21. The duration of the oscillations was approximately 10 sec (Figure 2(c2)).

Experiments revealed that hypoxia caused significant changes in the parameters of calcium activity on the 7th day of the posthypoxic period. We observed a decrease in the number of cells exhibiting spontaneous calcium activity (Figures 2(c1)–(c3)) (Sham: 95.40 ± 0.93 cells, hypoxia: 5.61 ± 2.32 cells; Sham: 4.41 ± 0.56 oscillations/min, hypoxia: 0.19 ± 0.07 oscillations/min; ANOVA *p* < 0.05, *N* = 12). In addition, the duration of the Ca^2+^ oscillations became stable on day 7 after hypoxia.

**Figure 3:**
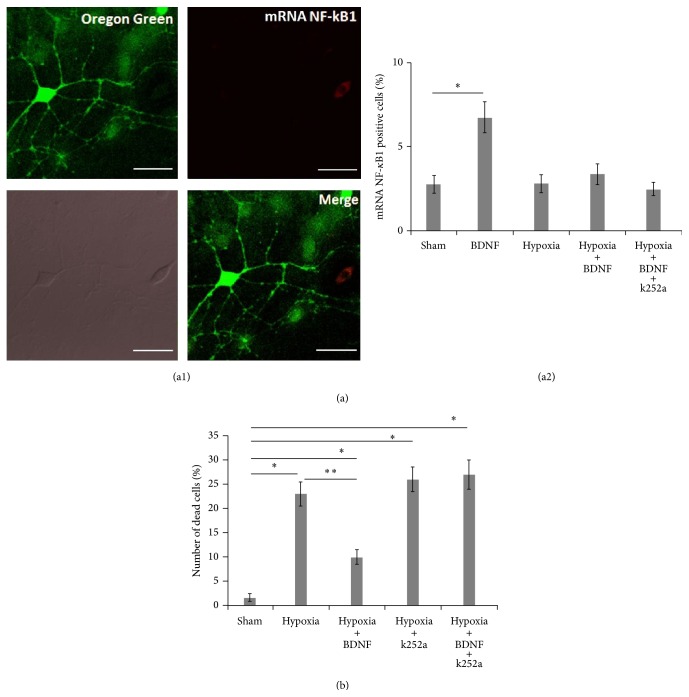
Viability determination and the detection of NF-*κ*B1 mRNA-positive cells in dissociated hippocampal cultures during the posthypoxic period. ((a1)-(a2)) Detection of NF-*κ*B1 mRNA-positive cells. (a1) An example of NF-*κ*B1-positive cells. Scalebar: 20 *μ*m; (a2) percentage of NF-*κ*B1 mRNA-positive cells on the first day after hypoxia. (b) Viability of cells after hypoxia; the number of dead cells in dissociated hippocampal cultures on day 7 after hypoxia (ANOVA *p* < 0.05, *N* = 12).

Moreover, there was an increase in the number of dead (propidium iodide-positive) cells after hypoxia (Figure 3(b)), and these cells did not exhibit calcium activity (Figure 2(b)-B3).

Application of BDNF (1 ng/mL) did not affect the duration of the Ca^2+^ oscillations. However, BDNF treatment did partially preserve the parameters of calcium activity in dissociated hippocampal cultures on day 7 of the posthypoxic period (Figures 2(c1)–(c3)) (proportion of cells exhibiting calcium activity: hypoxia: 5.61 ± 2.32 cells, hypoxia + BDNF: 33.87 ± 10.19 cells; hypoxia: 0.19 ± 0.07 oscillations/min, hypoxia + BDNF: 1.79 ± 0.36 oscillations/min; ANOVA *p* < 0.05, *N* = 12).

Next, we evaluated NF-*κ*B1 mRNA levels using fluorescent RNA probes for intravital imaging. Under normal conditions, approximately 2.4% of cells in dissociated hippocampal cultures were positive for NF-*κ*B1 mRNA expression at DIV 14. There was an increase in the number of NF-*κ*B1-positive cells the day after BDNF (1 ng/mL) application (Figure 3(a2)) (Sham: 2.76 ± 0.52 cells; BDNF: 6.75 ± 0.91 cells; ANOVA, *p* < 0.05, *N* = 9). However, a similar increase was not observed in the hypoxia + BDNF group after hypoxia (Sham: 2.76 ± 0.52 cells, hypoxia + BDNF: 3.36 ± 0.61 cells; ANOVA *p* < 0.05, *N* = 9).

In addition, hypoxia did not affect the number of NF-*κ*B1-positive cells, and there was no correlation between NF-*κ*B1-positive cells and spontaneous calcium activity in the primary neuronal cultures. At the same time, we found an increase in the number of dead cells on day 7 after hypoxia modeling (Figure 3(a2)) (Sham: 1.59 ± 0.76 cells, hypoxia: 23 ± 2.23 cells; ANOVA *p* < 0.05, *N* = 9).

We had established a reduction in the number of dead cells in dissociated hippocampal cultures following BDNF application (1 ng/mL). Application of the TrkB receptor antagonist increased the number of dead cells to a level similar to that observed in the “hypoxia” group (27.31 ± 3.21 cells).

## 4. Discussion

Hypoxia is considered to be one of the main factors involved in ischemic brain damage. The pathological reactions triggered by oxygen starvation are associated with an uncoupling of oxidative phosphorylation, disturbances in cellular energy levels, and the activation of free radical processes that stimulate apoptosis. The negative effects of oxygen deficiency are particularly important in the nervous system, where the loss of individual network elements can cause irreversible damage to larger functional units. Therefore, the ability of brain cells to protect themselves from oxygen starvation is one of the most important topics in modern neuroscience. These data demonstrate that BDNF has strong antihypoxic and neuroprotective properties. This protein can actively alter neuronal metabolism in adult brain. BDNF expression increases during sensory stimulation of neural networks in the brain [2, 23–28]. Moreover,* in vivo* experiments have shown that the expression of BDNF and TrkB mRNAs are affected in stress model [29]. In particular, there are significant increases in the mRNA and protein levels of BDNF under acute stress conditions. In contrast, the expression of BDNF mRNA and protein was reduced and the expression of TrkB mRNA was increased under chronic stress conditions [30].

It is known that in the nervous system BDNF is associated with two types of membrane receptors: the low-affinity nerve growth factor receptor (LNGFR, also known as p75) and the high-affinity tyrosine-kinase receptor B (TrkB) [12]. The binding strengths of these receptors for BDNF are different. Our experiments revealed that the antihypoxic (i.e., the viability of dissociated hippocampal cells) and neuroprotective (i.e., the preservation of spontaneous bioelectrical and calcium activity) properties of BDNF are associated with interactions between BDNF and the TrkB receptor. We demonstrated that blocking TrkB completely eliminated the positive effects of preventative BDNF treatment. Moreover, application of TrkB receptor antagonist caused more obvious destruction of neuronal networks compared with “hypoxia” group. This phenomenon is associated with inability endogenous BDNF binding with TrkB receptors following application of k252a. We hypothesized that the positive effects of BDNF during hypoxia are associated with the activation of NF-*κ*B1. NF-*κ*B1 family proteins play a special role in the inhibition of apoptosis. It is thought that the activation of NF-*κ*B in combination with a number of other factors (e.g., c-jun and cIAP1) serves as the primary anti apoptotic effector [31]. This research demonstrates that BDNF application stimulates NF-*κ*B1 mRNA synthesis in dissociated hippocampal cells under normal conditions. However, an increase NF-*κ*B synthesis in control group as well as in cultures with BDNF addition during hypoxia was not detected. Moreover, antagonist of TrkB did not alter NF-*κ*B1 mRNA synthesis.

## 5. Conclusion

The carried out experiments revealed acute hypoxia to cause irreversible changes in the functional bioelectrical and calcium network activity and reduces the viability of cells in dissociated hippocampal cultures. BDNF application contributed to the protection of cells from hypoxia. The antihypoxic and neuroprotective properties of BDNF are realized though binding with the TrkB receptor. Our experiments show that BDNF is involved in maintaining NF-*κ*B1 levels in dissociated hippocampal cultures. However, we suppose the neuroprotective effect of this neurotrophin does not depend on NF-*κ*B1 activation.
